# Endoscopic Removal of a Magnet Retained in the Stomach for Two Years: A Case Report and Literature Review

**DOI:** 10.7759/cureus.80562

**Published:** 2025-03-14

**Authors:** Hatem Ahmed, Sameh Gomaa, Imad Alabdul Razzak, Mohamad Talal Basrak

**Affiliations:** 1 Internal Medicine, Tower Health Medical Group, Phoenixville, USA

**Keywords:** endoscopic foreign body removal, foreign body ingestion, gastric outlet obstruction, magnet ingestion, magnet-retained

## Abstract

Foreign body ingestion (FBI) is a common clinical presentation, with most cases occurring in children. While accidental FBI in adults is less frequent, it is often associated with psychiatric conditions or substance use disorders. Magnet ingestion is particularly concerning due to the risk of pressure necrosis, perforation, and fistula formation, yet reports of magnet retention in adults are exceedingly rare.

We describe a 45-year-old male with a history of depression and substance use disorder who presented with a three-week history of progressive abdominal pain, nausea, bloating, and foul-smelling belching. Imaging revealed a radiopaque foreign body in the distal gastric antrum, later identified as a 2.5 cm metallic disc-shaped magnet. Notably, a prior CT scan performed two years earlier had documented the same object, which the patient had presumed to be a dental filling and expected to pass spontaneously. Esophagogastroduodenoscopy (EGD) successfully retrieved the magnet using a Roth net, with immediate symptom resolution.

This case is unique due to the prolonged asymptomatic retention of a magnet for two years before the onset of gastric outlet obstruction symptoms. While most foreign bodies pass spontaneously, endoscopic retrieval is warranted for magnets to prevent potential complications. The prolonged retention without perforation or obstruction highlights the role of anatomic location and object characteristics in determining outcomes. To our knowledge, this is the first reported case of successful endoscopic removal of a long-retained magnet in an adult using a Roth net.

This case underscores the importance of considering FBI in the differential diagnosis of unexplained gastrointestinal symptoms, particularly in high-risk patients. Early recognition and intervention are crucial to prevent severe complications. Endoscopic removal remains a safe and effective strategy even for delayed presentations, emphasizing the need for clinical vigilance in cases of unwitnessed FBI.

## Introduction

Foreign body ingestion (FBI) is a common medical emergency, with more than 100,000 cases reported annually in the United States, the majority occurring in children [1-4]. Globally, the incidence of FBI has significantly increased over the past three decades, with an estimated 66 million cases reported in 2019 [5]. FBI refers to the unintentional or intentional swallowing of objects or substances not naturally present in the body [5]. It is a frequent cause of emergency room visits for both children and adults. While most ingested foreign bodies pass spontaneously, some may become retained, leading to complications depending on their characteristics. High-risk populations include children, the elderly, individuals with psychiatric illnesses, and those with substance use disorders or alcohol intoxication [6,7].

Symptoms of FBI vary widely, ranging from epigastric pain, vomiting, and dysphagia to being completely asymptomatic in approximately one-third of cases [8]. Magnet ingestion, whether accidental or intentional, is particularly concerning, as multiple ingested magnets can attract each other across bowel walls, leading to pressure necrosis, ulceration, and mucosal indentation. These effects can develop within as little as eight hours, potentially resulting in bowel perforation and fistula formation [9].

To the best of our knowledge, reports of magnet ingestion in adults are limited. Here, we present the case of a 45-year-old male with a history of depression and substance use disorder who developed abdominal pain secondary to magnet ingestion after retaining the magnet for two years without symptoms.

## Case presentation

A 45-year-old male with a medical history of depressive disorder, managed with sertraline 25 mg daily, and hepatitis C, successfully treated, presented to the emergency department with a three-week history of abdominal pain, bloating, nausea, foul-smelling belching, and decreased appetite. He reported postprandial diarrhea during this period and experienced a single episode of greenish, non-bloody vomiting one day prior to presentation, prompting him to seek medical care. The patient denied fever, chest pain, shortness of breath, unintentional weight loss, gastrointestinal bleeding, recent travel, or antibiotic use. He had no prior history of abdominal surgeries or endoscopies and no family history of gastrointestinal disorders. His social history was significant for methamphetamine and tobacco use. He worked as a mechanic and reported occasionally consuming food at his workplace.

On examination, the patient was afebrile, normotensive, and maintaining oxygen saturation on room air but was tachycardic, with a heart rate of 112 beats per minute. He was alert and oriented. His abdomen was soft, with tenderness in the left upper quadrant but no distension. Bowel sounds were normal, and the remainder of his physical examination was unremarkable.

Initial laboratory studies, including a complete blood count (CBC), comprehensive metabolic panel (CMP), lipase, and urinalysis (UA), were within normal limits, except for mild hypercalcemia, hypomagnesemia, and hypokalemia, as shown in Table 1.

**Table 1:** Laboratory results of the patient *Abnormal laboratory value outside the reference range BUN, blood urea nitrogen; AST, aspartate aminotransferase; ALT, alanine transaminase; Alk Phos, alkaline phosphatase

Variables	Value	Reference range
Sodium	139	136-145 mmol/L
Potassium	3.4*	3.5-5.1 mmol/L
Chloride	107	100-110 mmol/L
BUN	9	9-23 mg/dL
Creatinine	0.87	0.73-1.18 mg/dL
Glucose	101*	74-99 mg/dL
Calcium	10.7*	8.7-10.4 mg/dL
Magnesium	1.5*	1.6-2.6 mg/dL
Lipase	23	12-53 IU/L
AST	21	34 IU/L
ALT	16	10-49 IU/L
Alk Phos	84	46-116 IU/L
White blood cells	8.6	4-11 × 10^3^/uL
Hemoglobin	14.2	12.7-18.0 g/dL
Platelets	217	140-400 × 10^3^/uL

**Figure 1:**
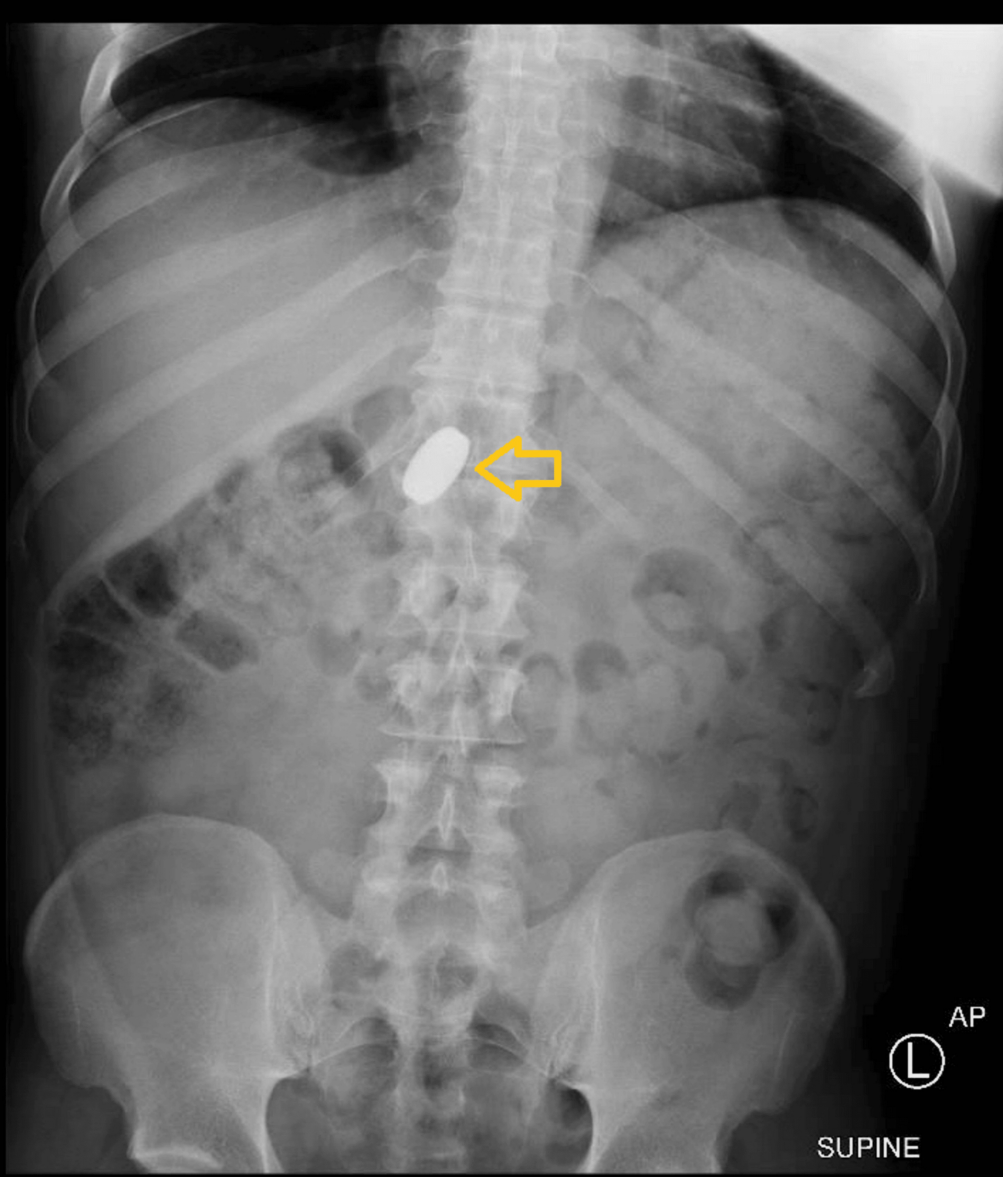
Anteroposterior supine view of the abdominal X-ray An abdominal X-ray showing a radiodense foreign body at T12/L1, consistent with an ingested metallic disc in the gastric antrum/pylorus.

A plain abdominal X-ray (Figure 1) revealed a radiodense foreign body just to the right of midline at the level of T12/L1, consistent with an ingested metallic disc or ring in the region of the gastric antrum/pylorus. The bowel gas pattern was unremarkable, with a moderate stool burden in the colon.

**Figure 2:**
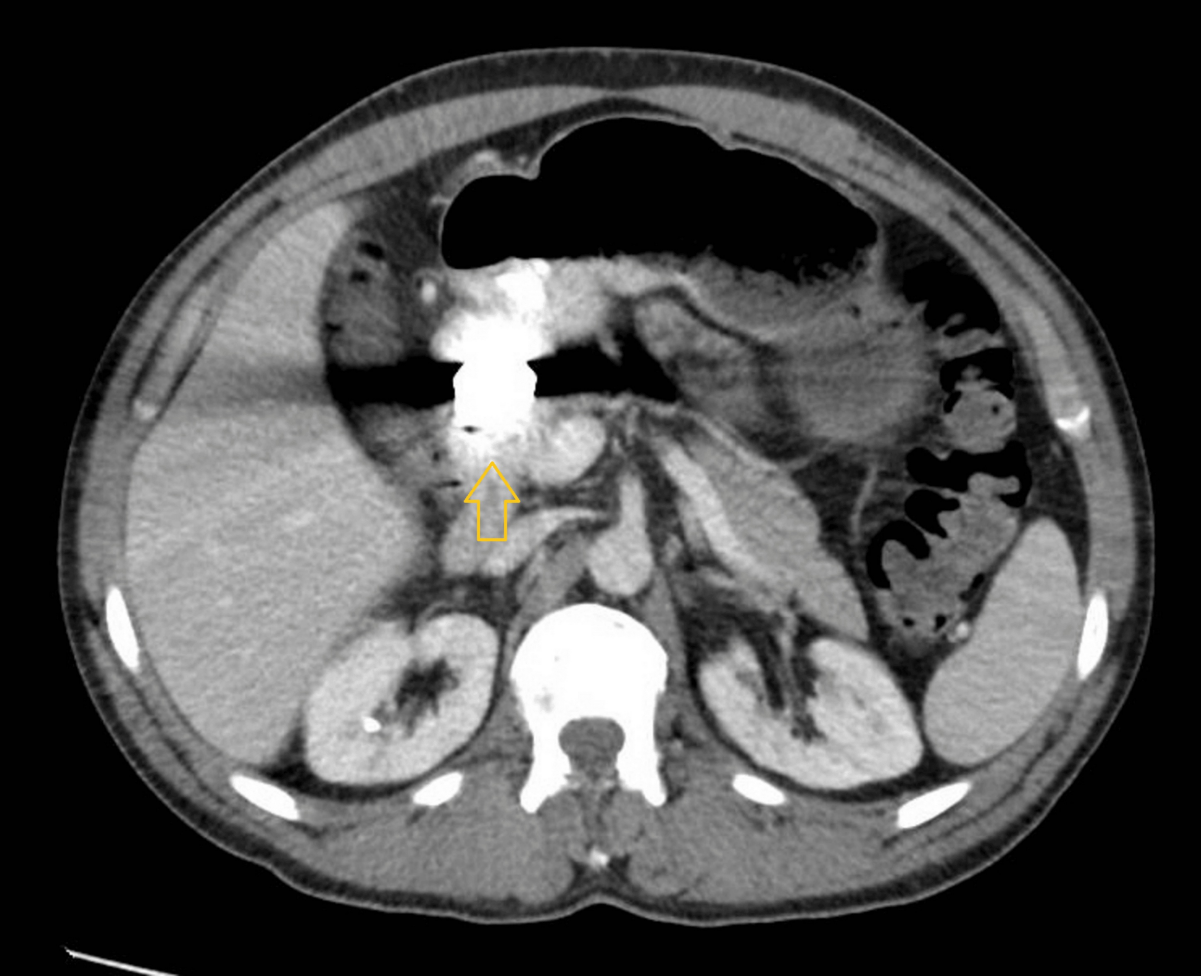
Axial view of the CT scan of the abdomen and pelvis Axial contrast-enhanced CT scan of the abdomen and pelvis showing a 2.5 cm disc-shaped metallic foreign body visible in the distal gastric antrum.

**Figure 3:**
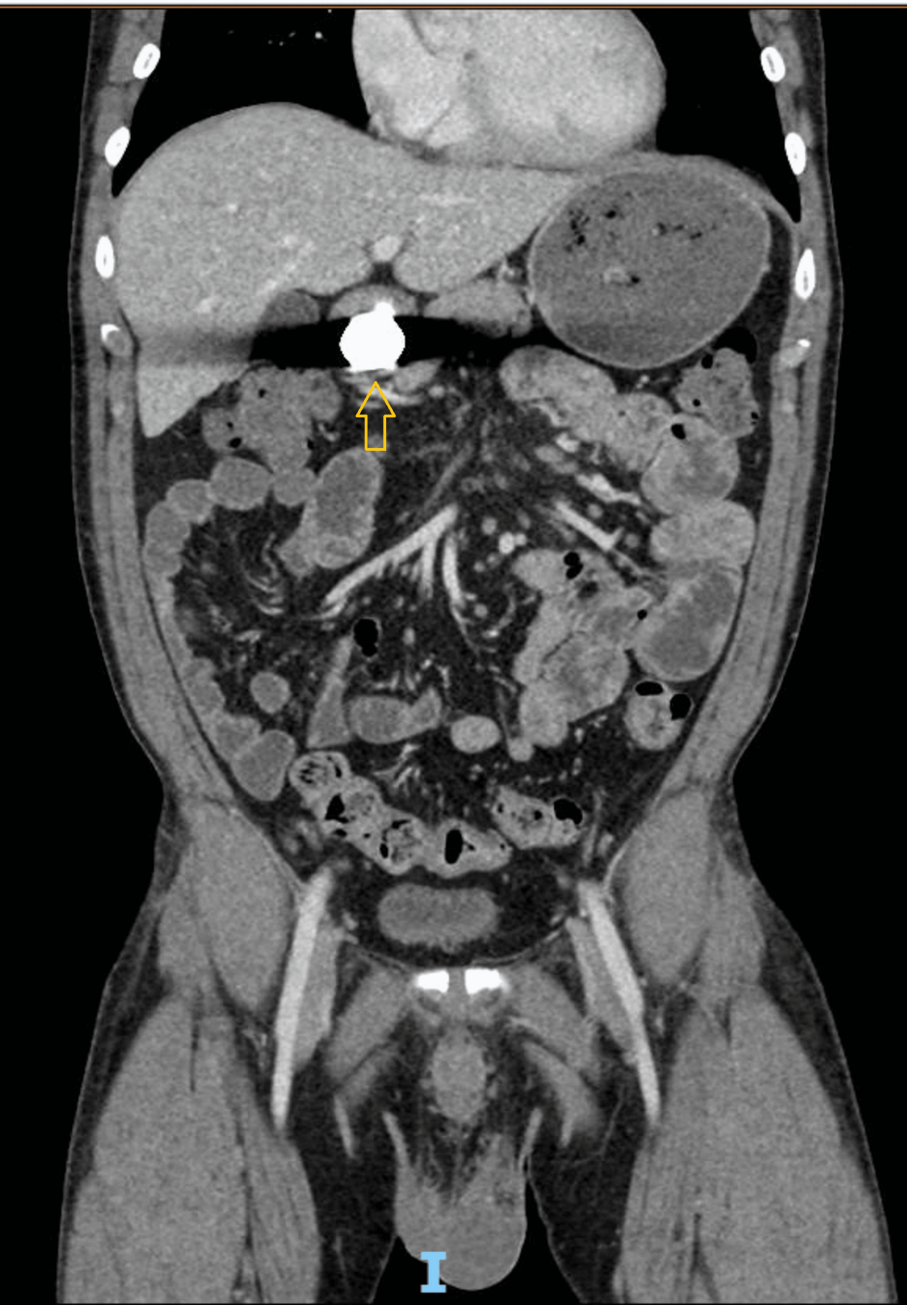
Coronal view of the CT scan of the abdomen and pelvis Coronal contrast-enhanced CT scan of the abdomen and pelvis demonstrating a retained metallic foreign body in the distal gastric antrum.

A contrast-enhanced CT scan of the abdomen and pelvis (Figures 2, 3) demonstrated the following findings: gastric distension with fluid, ingested food, and gas, along with residual bismuth subsalicylate in the stomach; a 2.5 cm disc-shaped metallic foreign body located in the distal gastric antrum (or, less likely, the proximal duodenum); mildly dilated loops of mid-small bowel with fluid but no evidence of bowel obstruction; and semisolid fecal material and gas in the colon without wall thickening or signs of colitis.

**Figure 4:**
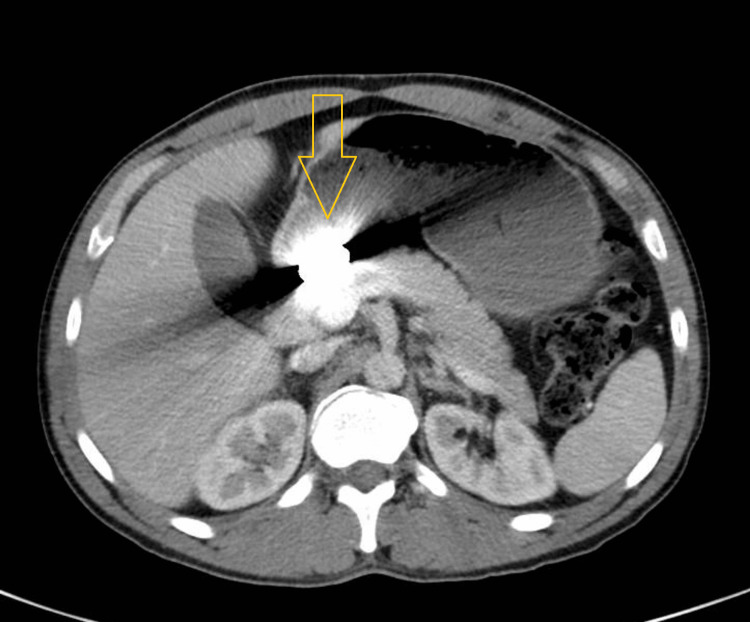
Axial view of the CT scan of the abdomen and pelvis Axial contrast-enhanced CT scan of the abdomen and pelvis, obtained two years prior to this presentation, showing a metallic foreign body in the stomach with beam hardening artifacts.

**Figure 5:**
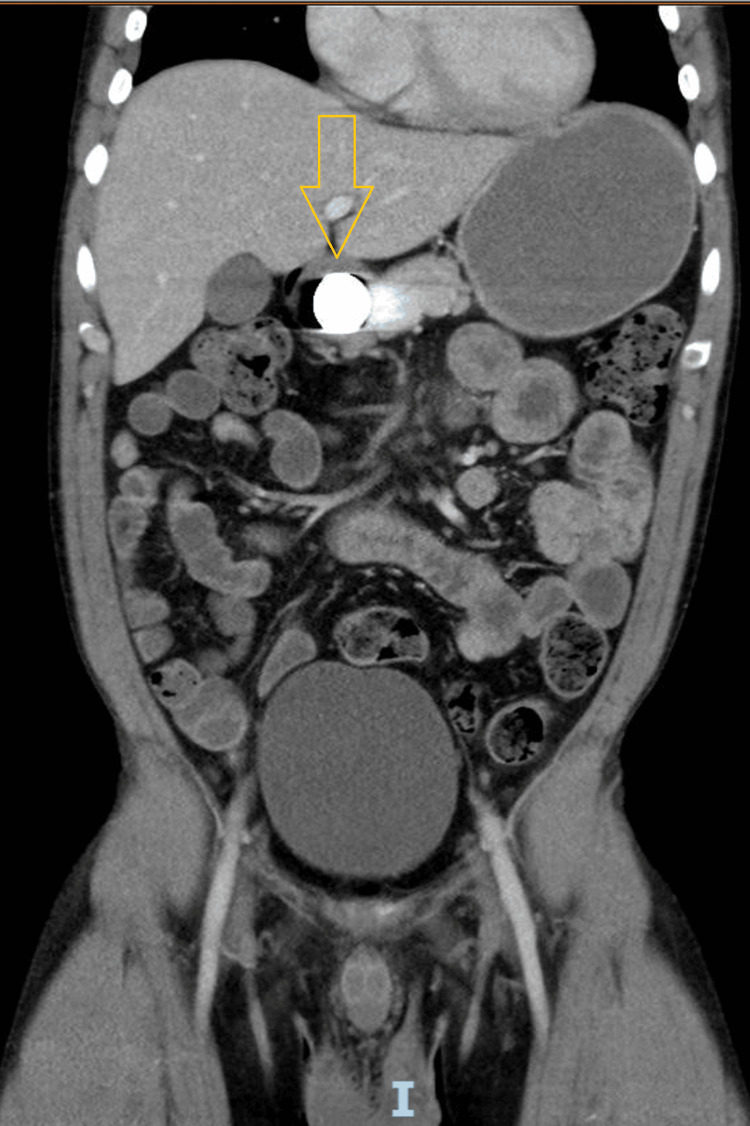
Coronal view of the CT scan of the abdomen and pelvis Coronal contrast-enhanced CT scan of the abdomen and pelvis, performed two years before this presentation, demonstrating a retained metallic foreign body in the stomach.

The patient reported a prior episode of abdominal discomfort two years before this presentation, during which he underwent a contrast-enhanced CT scan of the abdomen and pelvis (Figures 4, 5). The scan identified a metallic foreign body in the stomach with beam hardening artifacts, along with 1-2 mm non-obstructing bilateral renal calculi. At that time, he left the hospital against medical advice, assuming he had accidentally swallowed a dental filling that would pass spontaneously. He denied any interval dental work, intentional FBI, or additional symptoms between that episode and his current presentation.

The patient was admitted with a diagnosis of gastric outlet obstruction secondary to a retained foreign body. The gastroenterology team was consulted and recommended keeping the patient nothing by mouth (NPO), inserting a nasogastric tube (NGT) for gastric decompression, and administering intravenous fluids, with plans to perform esophagogastroduodenoscopy (EGD) the following day for foreign body extraction.

**Figure 6:**
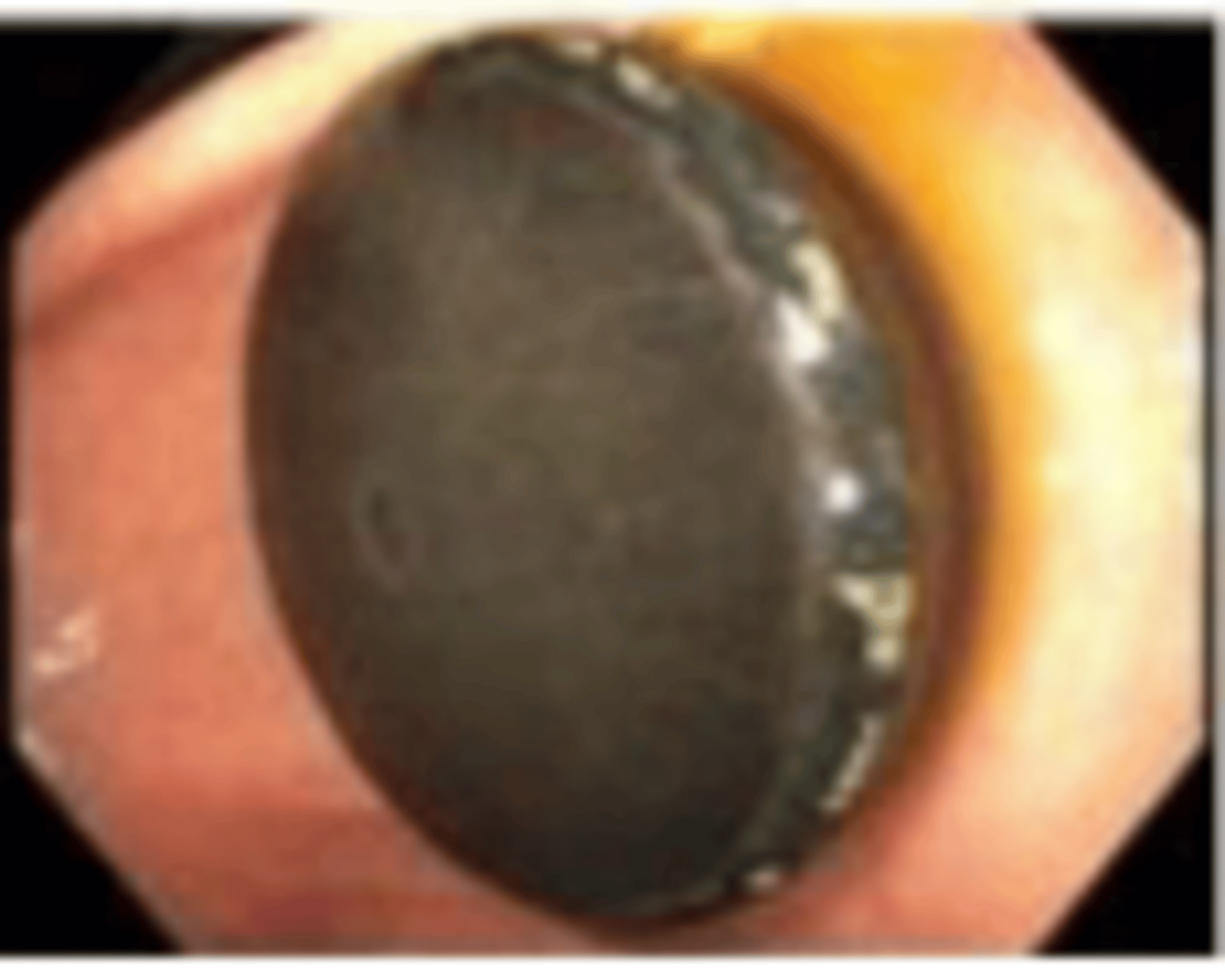
Endoscopic view of distal stomach Endoscopic view of a floating, black, disc-shaped foreign body in the distal stomach.

The patient underwent EGD the next day under general anesthesia with endotracheal intubation for airway protection. The procedure revealed a floating, black, disc-shaped foreign body in the distal stomach (Figure 6). Using a Roth net, the object was successfully removed. The foreign body was identified as a magnet. The remainder of the esophagus, stomach, and proximal small bowel appeared normal.

The foreign body was sent for analysis and described as a 2.4 × 2.4 × 0.6 cm gray-black metallic disc with magnetization and a central, slightly depressed area measuring 0.6 cm in diameter. No adherent soft tissue was identified. Post-procedure, the patient tolerated a regular diet within two hours and was discharged home in stable condition.

## Discussion

Early identification and appropriate management of FBI are crucial. Determining the location and characteristics of the ingested object is time-sensitive. Chest and abdominal X-rays are typically the first-line diagnostic tools, as they effectively detect radiopaque objects. However, CT imaging may be necessary for radiolucent foreign bodies, particularly when clinical suspicion remains high despite negative radiographs [8].

Although 80% of ingested foreign bodies pass spontaneously, endoscopic intervention is required in approximately 20% of cases, while surgical intervention is necessary in less than 1%. Conservative management is often appropriate for blunt, short (<6 cm), and narrow (<2.5 cm diameter) objects, especially if they have passed the pylorus, with spontaneous passage typically occurring within four to six days. However, endoscopic removal within 24 hours is indicated for sharp or pointed objects, batteries, magnets, and objects >2.5 cm in diameter or >6 cm in length. Emergency endoscopy is warranted for cases of complete esophageal obstruction or when there is a risk of aspiration. Surgical intervention is reserved for foreign bodies that remain in the distal duodenum for over a week or in cases of complications such as perforation [10].

According to the American Society for Gastrointestinal Endoscopy (ASGE) guidelines, all magnets within endoscopic reach should be urgently removed to prevent complications. For magnets beyond endoscopic reach, close monitoring and surgical consultation are recommended in cases of non-progression [11].

The management strategy depends on the number and location of the magnets. A single magnet can often be managed conservatively, provided there are no symptoms or signs of obstruction. However, multiple magnets or a single magnet with a metallic object necessitate urgent endoscopic or surgical removal, particularly for larger objects (>20-25 mm). If a single magnet is located in the esophagus or stomach, endoscopic retrieval is preferred, especially if the patient is at risk of further ingestion, if the magnet fails to progress, or if symptoms develop. In cases where multiple magnets or a single magnet with a metallic object are present in the esophagus or stomach, endoscopic removal should be performed within 12 hours; if removal is not feasible within this timeframe, surgical intervention is warranted after 12 hours.

If conservative management is pursued, serial X-rays should be obtained in the outpatient setting to monitor progression. In cases of delayed progression, polyethylene glycol or other laxatives may be utilized to facilitate passage. When the magnet has moved beyond the stomach and the patient is asymptomatic with no evidence of complications, enteroscopic or colonoscopic removal may be considered. Otherwise, serial X-rays combined with laxatives and close monitoring may be an alternative approach. If the patient develops worsening symptoms or complications arise, surgical intervention becomes necessary [4,9,12].

To the best of our knowledge, reports of magnet ingestion in adults are limited, with varying clinical presentations and management approaches. Table 2 summarizes previously reported cases, highlighting the number of magnets ingested, associated complications, and treatment outcomes.

**Table 2:** Reported cases of magnet ingestion in adults EGD, esophagogastroduodenoscopy

Age (years)	Number of magnets ingested	Complications	Treatment outcome	Reference
18	Multiple + nail	Pressure necrosis of transverse colon, jejunal perforation	Exploratory laparotomy; magnets removed; stomach and jejunum surgically repaired	[13]
59	Two	Incidental discovery; remained in stomach for years	EGD removal using rat-tooth forceps; no complications	[7]
28	Multiple	Cecal perforation, intra-abdominal infection	Laparotomy; four magnets removed, one passed spontaneously	[14]
21	Two	Intestinal obstruction, strangulation, and jejuno-ileal fistula	Laparotomy; intestinal segments devolvulated and perforations sutured; successful recovery	[15]

This case describes a middle-aged male who remained asymptomatic for two years following the ingestion of a 2.5 cm disc-shaped metallic object, later identified as a magnet. Despite its prolonged retention, the foreign body did not cause perforation, obstruction, or ischemia, which are commonly associated with magnet ingestion. The patient presented only when gastric outlet obstruction symptoms developed, prompting further investigation.

Endoscopic retrieval was successfully performed using a Roth net, a technique not previously documented in adult cases of magnet ingestion. The absence of severe complications in this case was likely due to the object’s shape and location, which prevented entrapment or attraction to adjacent structures.

While delayed diagnoses of FBI are not uncommon, asymptomatic retention for such a prolonged period is rare. Cases of foreign bodies persisting undetected for years, or even decades, have been reported [16], but magnets pose a unique risk due to their potential to cause internal damage via magnetic attraction. This case reinforces the importance of considering FBI in patients presenting with unexplained gastrointestinal symptoms, especially those with risk factors such as psychiatric conditions or substance use disorders.

## Conclusions

This case highlights that a single retained magnet can remain asymptomatic for years before causing symptoms. The absence of complications was likely due to its anatomical positioning and lack of interaction with other metallic objects. Retrospective imaging confirmed its prolonged retention, emphasizing the importance of imaging review in patients with a history of FBI.

Successful endoscopic removal using a Roth net demonstrates the safety and efficacy of this approach, even in delayed presentations. Early recognition and timely intervention are crucial in preventing serious complications, particularly in high-risk populations.
